# Minimally invasive excision and reconstruction of Achilles tendon xanthoma using free autologous semitendinosus tendon transfer: a surgical technique

**DOI:** 10.1186/s13018-023-03757-x

**Published:** 2023-04-04

**Authors:** Francesco Oliva, Emanuela Marsilio, Federica Mastrodonato, Filippo Migliorini, Nicola Maffulli

**Affiliations:** 1 Department of Medicine, Surgery and Dentistry, University of Salerno, 84081 Baronissi, Salerno, Italy; 2 Department of Orthopaedic, Trauma, and Reconstructive Surgery, RWTH University Hospital, Pauwelsstraße 30, 52074 Aachen, Germany; 3 Department of Orthopaedic and Trauma Surgery, Eifelklinik St. Brigida, 52152 Simmerath, Germany; 4 School of Pharmacy and Bioengineering, Keele University Faculty of Medicine, ST4 7QB Stoke on Trent, England; 5 Barts and the London School of Medicine and Dentistry, Centre for Sports and Exercise Medicine, Mile End Hospital, Queen Mary University of London, E1 4DG London, England

**Keywords:** Xanthoma, Achilles tendon, Surgery, Tendon graft, Hypercholesterolemia

## Abstract

**Background:**

Tendon xanthomatosis is often associated with familial hypercholesterolemia, but it can also occur in other medical conditions. The Achilles tendon is the most common site of tendon xanthomas. Reconstruction of large defects after the xanthoma excision, can be challenging.

**Methods:**

We propose a novel technique for Achilles tendon reconstruction with the use of an ipsilateral autologous semitendinosus tendon graft. The technique consists of six steps.

**Results:**

This procedure has a low rate of complications and provides results that are at least comparable with those reported with other surgical approaches.

## Introduction

The term xanthoma derives from the Greek word “xanthos” (yellow) [1]. Tendon xanthomas are benign masses characterized by lipid deposition within the tendon structure, frequently associated with genetic factors; they may be single or bilateral [2]. Tendon xanthomas are often associated with coronary artery disease and a high risk of cardiovascular events [3, 4]. In addition to the Achilles tendons, the other tendons where xanthomas usually appear are the tendons of the hand (extensor tendons) and the elbow [5]. Xanthomas have also been reported in the plantar fascia, fascia and periosteum overlying the lower tibia, the peroneal tendons, the triceps tendon and the patellar tendon [6]. Mechanical stress and neovascularization are considered as predisposing factor for xanthomas growth [7]. In addition to tendinous xanthomas, several types of xanthomas can be found such as tuberous and eruptive xanthoma, eyelid xanthelasma and xanthoma planum [6]. Xanthomas are frequently associated with hyperlipidemia, and high levels of triglycerides and total cholesterol: within families affected by familial hypercholesterolemia and type II hyperlipoproteinemia Achilles tendon xanthomas are frequent, probably because of the low-density lipoprotein storage in the tendon [7]. Furthermore, the presence of endothelial cells and macrophages is the main factors contributing to the pathogenesis of tendon xanthomas [8]. Achilles tendon xanthomas may be detected by clinical examination and imaging studies. Clinical evaluation, including inspection and palpation of the Achilles tendon, plays a fundamental role to reach the diagnosis [5]; demonstrating painless soft tissue nodules or aching papules that can be detected within the tendon, most commonly in its distal portion [8, 9]. Achilles tendon xanthomas are usually accompanied by an increase in tendon size, caused not only by the intratendinous storage of lipids but also by the edema and inflammation of the area [5]. Xanthomas of the Achilles tendon can cause pain, swelling, loss of function and problems with footwear, and psychological problems related to the deformity of the affected limb [10]. Ultrasonography is considered the first-line imaging examination. Xanthomas are visualized as hypoechoic nodules or a diffuse heterogeneous abnormality of the tendon, with markedly increased anteroposterior diameter [11, 12]. Surgical treatment of Achilles xanthomas tendon is usually limited to patients who have severe enlargement of the tendon, causing pain or mobility problems [13]. Several drugs, such as Pravastatin and Lovastatin, can decrease the size of the xanthomas; but they recur when the medication is stopped [5, 14, 15]; have, but they recur when the medication is stopped [16, 17]. We propose a novel technique in which the Achilles tendon xanthoma is removed in its entirety, and the tendon defect reconstructed using an ipsilateral free semitendinosus tendon graft. This procedure has a low rate of complications and provides results that are at least comparable with those reported with other surgical approaches.

## Material and methods

The technique consists of six steps.

Step 1: Patient positioning.

Step 2: Incision, Semitendinosus tendon visualization and harvest.

Step 3: Achilles tendon insertion incision and xanthoma exposure.

Step 4: Xanthoma excision.

Step 5: Calcaneal osteotomy and tunnel drilling.

Step 5: Tunnel passage.

Step 6: Shuttling of the tendon graft in the calcaneal tunnel.

Step 7: Graft fixation with an interference screw and deep tissues and skin closure.

### Surgical procedure

**Fig. 1:**
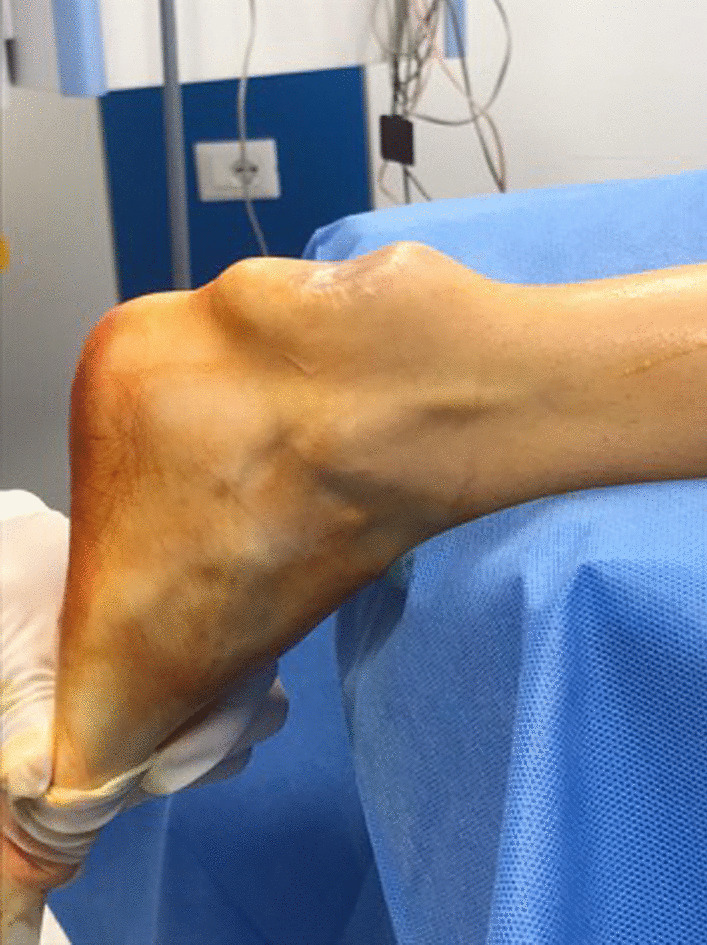
Pre-operative clinical photograph of the ankle showing the xanthoma of the Achilles tendon

With the patient prone under epidural, spinal or general anesthesia, a tourniquet is applied to the root of the thigh and inflated to 300 mmHg after exsanguination (Fig. 1). Skin preparation is performed in the usual fashion, and the sterile field is prepped.

**Fig. 2:**
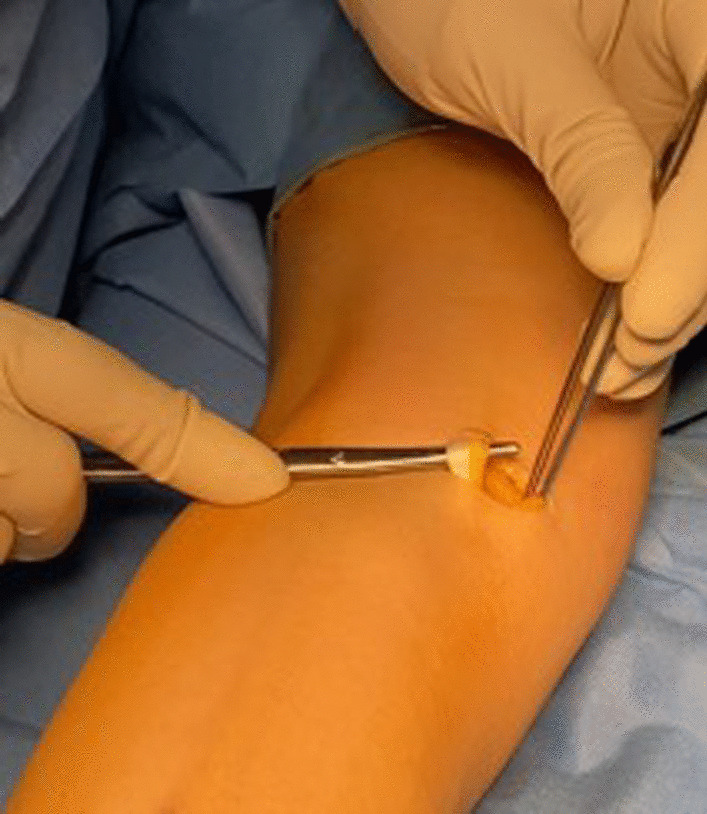
Transverse incision of about 2 cm on the medial aspect of the popliteal fossa. The semitendinosus tendon is visualized

**Fig. 3:**
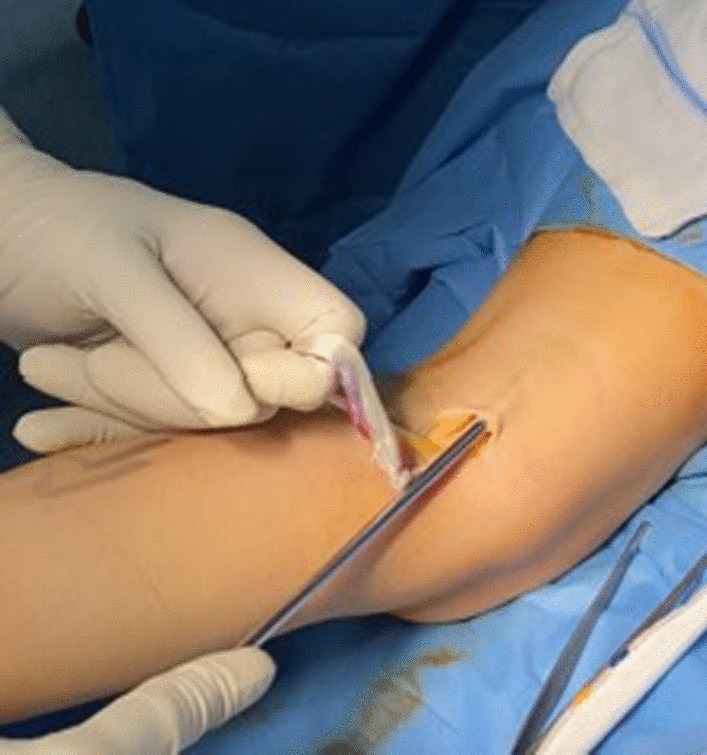
Harvest and preparation of semitendinosus tendon graft

Accurate palpation allows to identify the semitendinosus tendon in the postero-medial corner of the popliteal fossa. The tendon is harvested through a 2.5–3 cm transverse incision (Figs. 2 and 3).

**Fig. 4:**
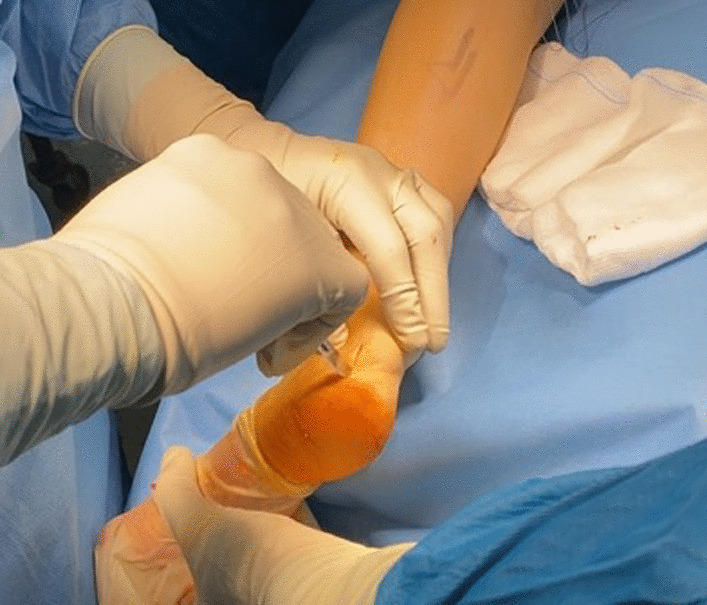
The postero-lateral incision at the Achilles tendon insertion

The Achilles tendon insertion is reached through a para-Achilles postero-lateral incision, the size of which may vary according to the size of the xanthoma (Fig. 4).

The proximal portion of the Achilles tendon is exposed through a para-Achilles postero-medial incision, and the xanthoma appears as a large yellowish white nodular mass involving the Achilles tendon.

**Fig. 5:**
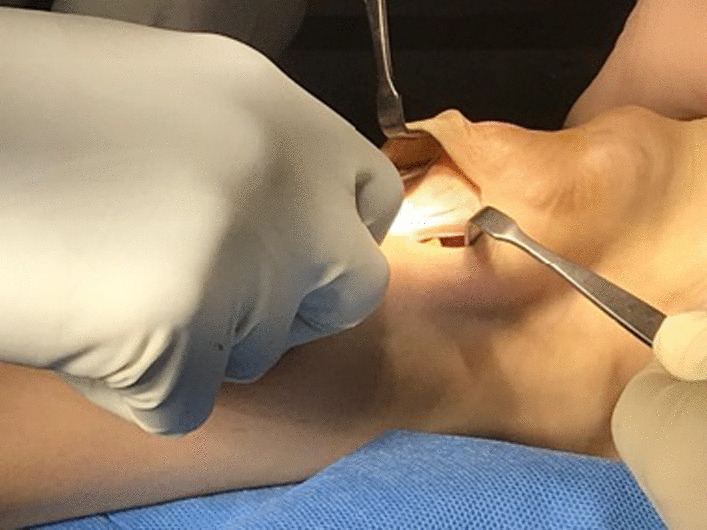
Removal of xanthoma adhesions

**Fig. 6:**
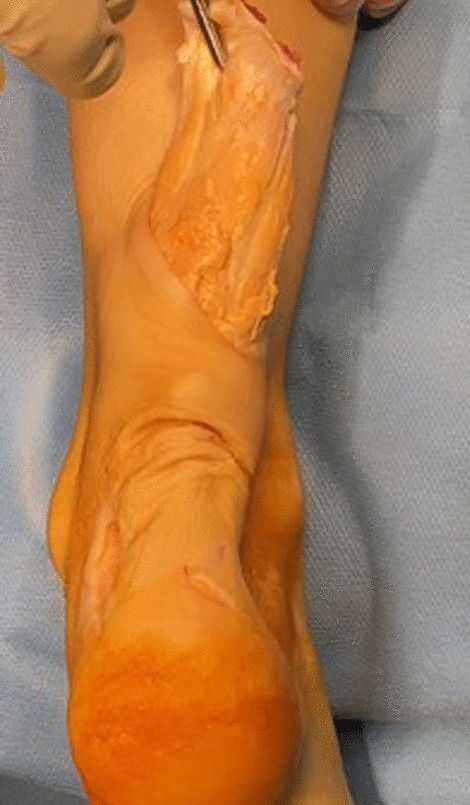
Excision of the Achilles tendon xanthoma

**Fig. 7:**
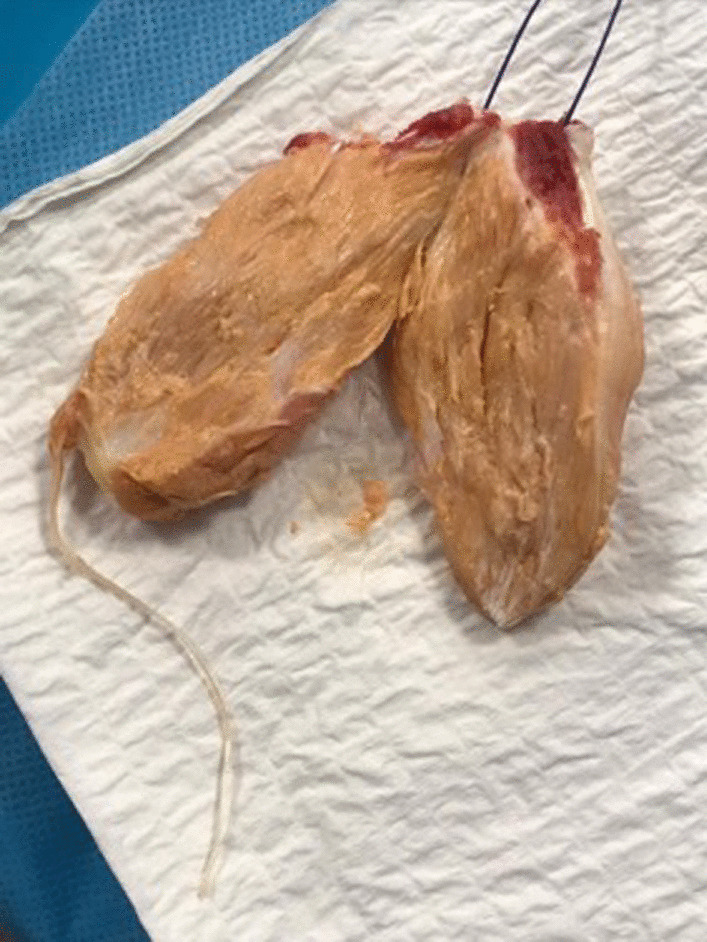
Section of the removed xanthoma

After detaching and removing the adherences (Fig. 5), the xanthoma can be excised (Figs. 6 and 7).

**Fig. 8:**
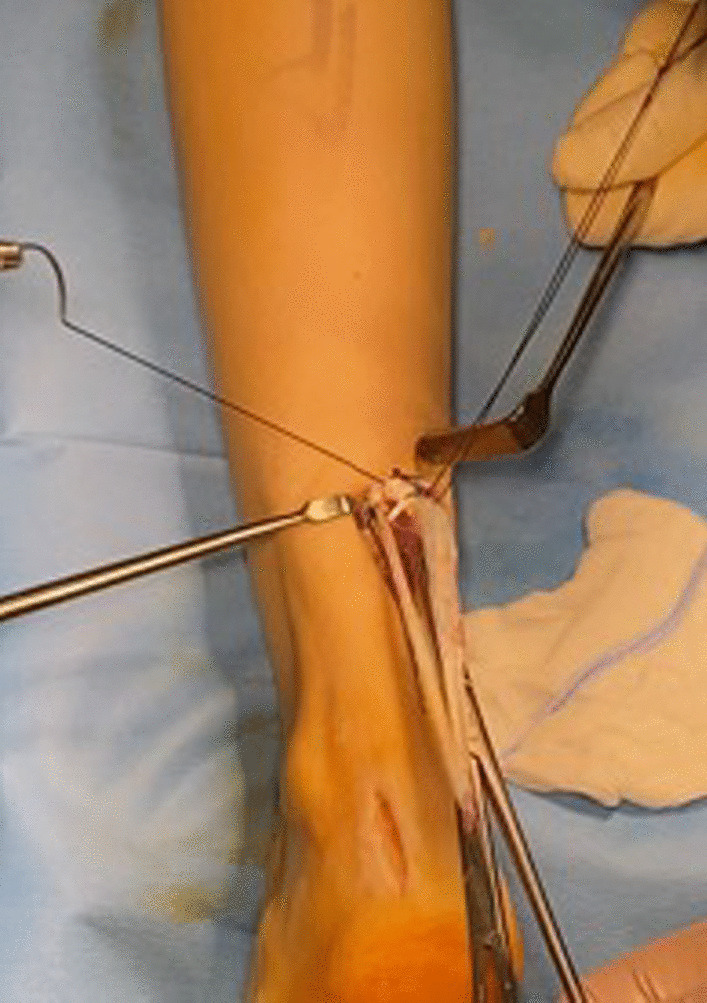
The extremities of the semitendinosus tendon are with 2 Vicryl (Polyglactin 910 Braided Resorbable Suture; Johnson & Johnson, Brussels, Belgium) in a whipstitch configuration, and passed transversely through the proximal stump of the Achilles tendon. The entry and exit points are secured with two Vicryl

After its removal, a large defect is present, which can be difficult to reconstruct from primary repair. Each end of the semitendinosus tendon is then prepared using 2 Vicryl (Polyglactin 910 Braided Resorbable Suture; Johnson & Johnson, Brussels, Belgium) with three or four passes in a whip stitch configuration (Fig. 8).

The proximal end of the Achilles tendon is mobilized and freed from the peritendinous adherences. An osteotomy of the posterior-superior corner of the calcaneus with a small sagittal saw is made, removing a “-slice-” of bone approximately 5 mm thick, without removing the distal tendon stump, exposing the insertion of the Achilles tendon on the calcaneus.

**Fig. 9:**
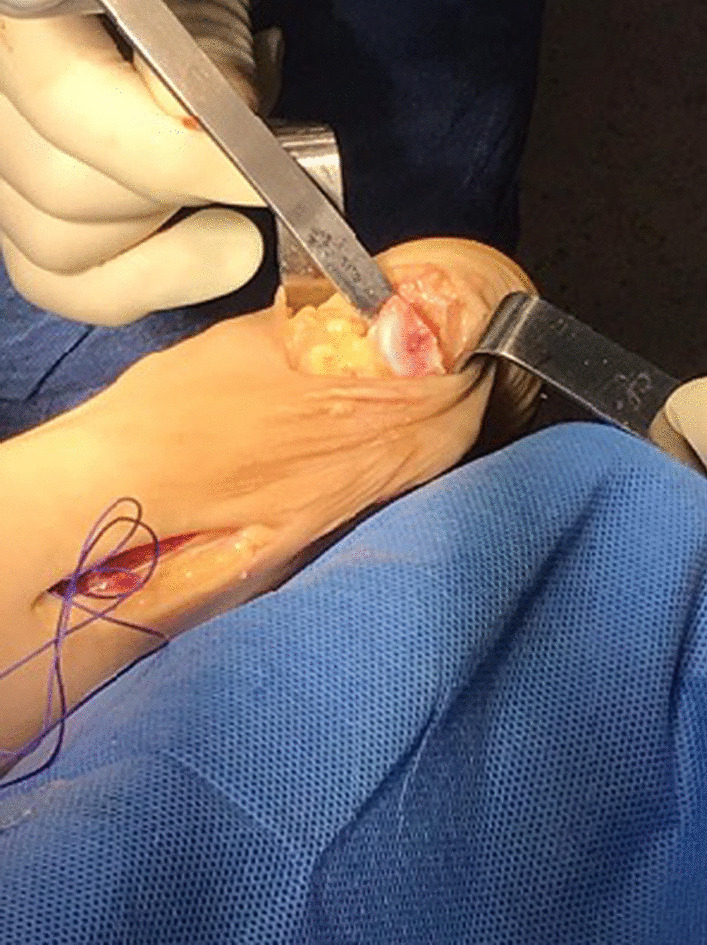
Calcaneus preparation, osteotomy, and tunnel drilling

The osteotomy is started just above the insertion of the Achilles tendon on the calcaneus and performed with an angle of approximately 45° to the long axis of the tendon (Fig. 9).

The calcaneus is prepared removing the posterosuperior corner and drilling a tunnel of an appropriate diameter just anterior to the insertion of the Achilles tendon on the calcaneus (Fig. 9).

The looped semitendinosus graft is passed transversely through the substance of the proximal stump of the Achilles tendon and sutured at both its entry and exit points with two Vicryl sutures to achieve adequate stability and prevent distal migration of the graft.

**Fig. 10:**
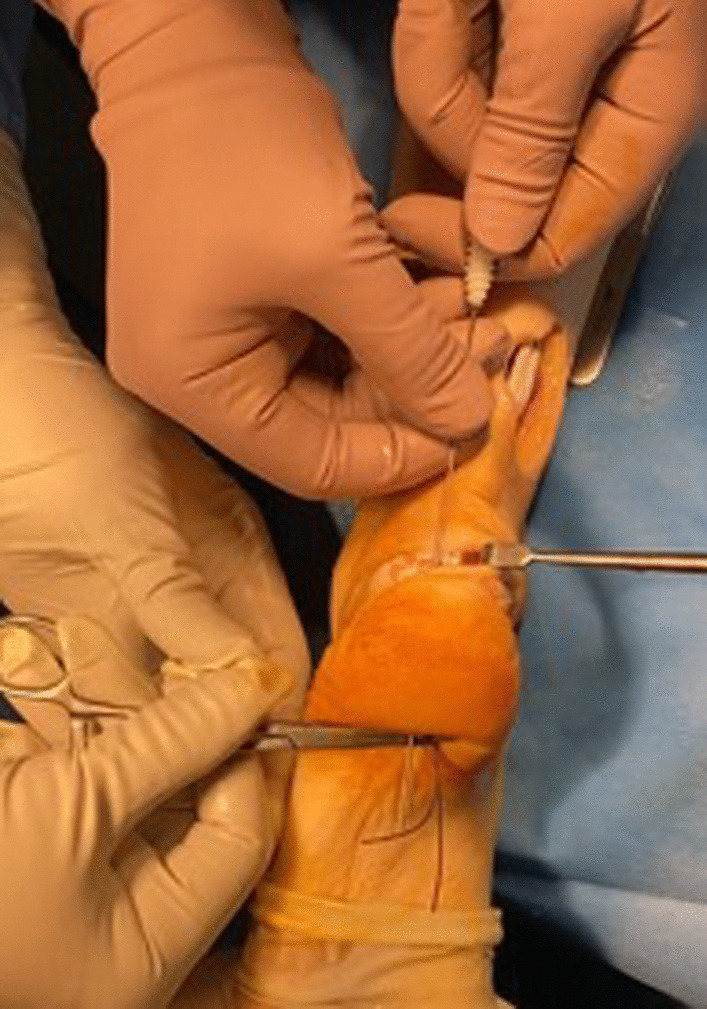
The two ends of the semitendinosus tendon are shuttles distally and in the calcaneus tunnel, and the tendon graft in secured to the calcaneus

**Fig. 11:**
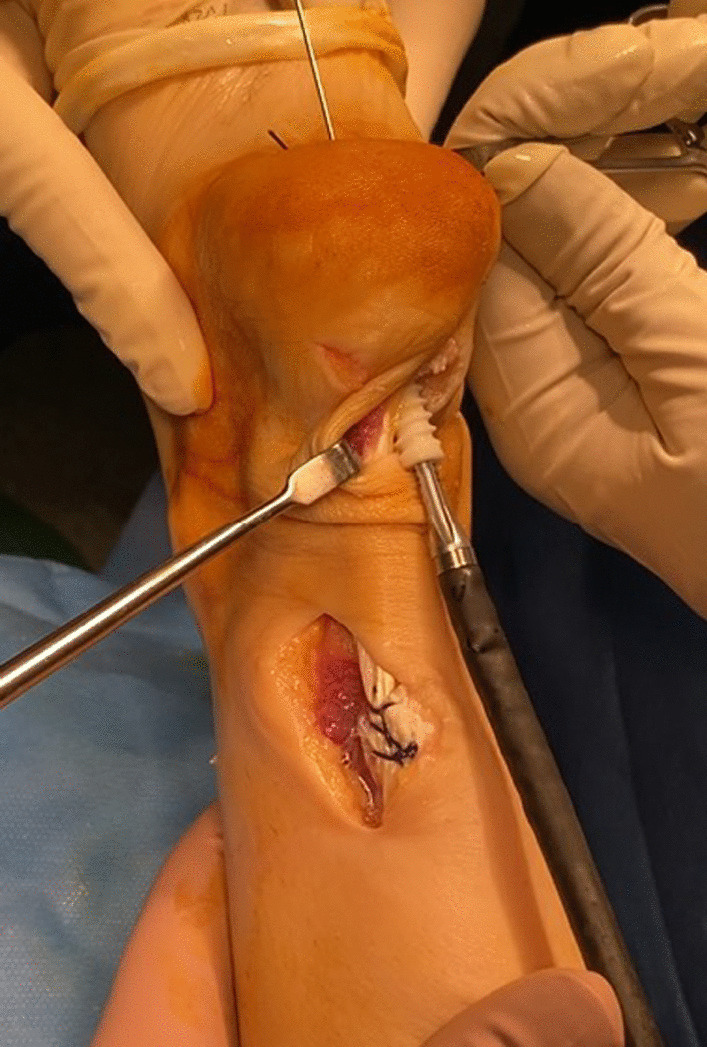
Fixation of the graft with an interference screw

The graft is passed through the subcutaneous tissue, shuttled through the calcaneus tunnel, and then secured to the calcaneus with an interference screw of appropriate diameter keeping the ankle in maximum plantar flexion. (Figs. 10 and 11) The final position and stability are verified, and the incisions are sutured in a started fashion. The patient is immobilized in a synthetic below the knee cast, leaving the metatarsal heads free.

### Post-operative protocol

After surgery, the leg is immobilized in a below the knee plaster cast with the foot in equinus leaving the metatarsal heads free. The patient can weight bear on the metatarsal heads with the use of elbow crutches immediately. After 3 weeks, the plaster cast is removed and an Aircast boot with five heel wedges is applied (XP Walker, DJO Ltd, Guilford, England, UK). Physiotherapy starts immediately afterward, focusing on proprioception, plantar flexion of the ankle, inversion, and eversion of the foot, avoiding dorsiflexion of the ankle and local massage [18, 19]. During this rehabilitation phase, patients are directed to weight bear as comfortable. Although full weight bearing is recommended, patients usually require a single crutch for two or three weeks [20, 21]. A heel wedge is removed at the end of the fourth week, and the boot is finally removed at the end of the eighth-tenth post-operative week [22]. The “GAIT” study group (German, American, Italian Tendon) established a post-operative protocol for the rehabilitation of Achilles tendon rupture after surgical reconstruction agreeing on a first period of non-weight bearing (2.3 weeks), and the first 4 weeks of plantarflexion, followed by concentric bilateral heel raises exercises from the 6^th^ week. Before 12 weeks exercises beyond neutral to restore the ROM, stretching, and eccentric exercises are not recommended, while the use of treadmill and heel pads to be placed in shoes seems to be useful for a full rehabilitation [23].

## Discussion

Tendon xanthomas are yellowish masses from macrophage deposits full of lipids [24]. Clinical examination and past medical family history can guide the diagnosis of xanthomatosis [25]. Although the usual treatment of Achilles tendon xanthoma is directed primarily toward lowering the level of serum cholesterol and managing the complications of coronary atherosclerosis, some patients require surgical management. Surgical removal is suggested in large tendon xanthomas that can limit joint motion and cause physical or psychological discomfort [6]. Several techniques of reconstruction of the Achilles tendon after xanthoma removal have been described. Samal et al. described bilateral reconstruction of the Achilles tendon with the use of the peroneus brevis tendon graft, giving a satisfactory outcome [2]. Senthil et al. described reconstruction of the Achilles tendon after removal of the xanthoma with a tensor fascia lata graft, with results comparable to the reconstruction performed by tendon-calcaneal allograft in terms of patients reported outcomes [9]. Moroney et al. described reconstruction of the Achilles tendon after removal of the xanthoma using a flexor hallucis longus tendon transfer and a Bosworth turndown flap. This combination avoids donor site morbidity, minimizes the risk of infection, and uses locally material available for reconstruction. Furthermore, the distal location of the muscle belly of the flexor halluces longus provides improved vascularization to the reconstructed site [26]. It requires wide exposure of the tendon and gastrocnemius to allow fashioning of the turndown flap. Saraf et al. used a flap with distal base modeled from the remainder proximal tendon and aponeurosis of the gastrocnemius. After 2 years of follow-up, patients were able to stand on tiptoe and the ankle was stable with acceptable cosmetic appearance [27]. Other reconstruction techniques have been described, including VY advancement flaps, proximal gastrocnemius/soleus turndown flaps, cadaveric allografts and synthetic grafts [28–37]. No technique is considered as gold standard for the surgical management of large defects of the Achilles tendon. Our study offers a novel and reproducible less invasive technique of Achilles tendon reconstruction with a semitendinosus tendon graft after removal of large xanthomas.

## Conclusion

Large xanthomas of the Achilles Tendon are uncommon. One of the most common problems is incomplete removal of the lesion that predisposes for recurrence. Reconstruction of the defect after complete excision remains challenging. Several reconstruction options are available after removal of an Achilles tendon xanthoma, and the use of an ipsilateral semitendinosus tendon autograft is a safe reproducible surgical option.

## Data Availability

The datasets generated during and/or analyzed during the current study are available throughout the manuscript.
